# Early feeding and risk of Juvenile idiopathic arthritis: a case control study in a prospective birth cohort

**DOI:** 10.1186/s12969-017-0175-z

**Published:** 2017-05-26

**Authors:** Erik Kindgren, Mats Fredrikson, Johnny Ludvigsson

**Affiliations:** 1 Department of Pediatrics, Västervik Hospital, Västervik, Sweden; 2 Division of Pediatrics, Department of Clinical and Experimental Medicine, Linköping University, Linköping, Sweden; 3 Division of Occupational and Environmental Medicine, Department of Clinical and Experimental Medicine, Linköping University, Linköping, Sweden; 4 Forum Östergötland, Faculty of Medicine, Linköping University, Linköping, Sweden

**Keywords:** Juvenile idiopathic arthritis (JIA), Arthritis, Epidemiology, Autoimmunity, Nutrition, Reactive arthritis, Breastfeeding, Rheumatic disease

## Abstract

**Background:**

Juvenile idiopathic arthritis (JIA) is considered to be an autoimmune disease, but the etiology is unknown. We decided to study the influence of early nutrition on later development of JIA.

**Methods:**

All parents with children born between October 1, 1997 and October 1, 1999 in Southeast Sweden were asked to participate in the ABIS prospective cohort study (All Babies in Southeast Sweden), At 1 year, questionnaires with information on breastfeeding and introduction of foods were completed by 10,565 families. We identified 32 children with JIA and 111 children with non-chronic arthritis with completed questionnaires after delivery and after 1 year. A multivariable logistic regression model, adjusted for relevant factors, was performed to calculate the association between JIA and feeding during the first year of life.

**Results:**

﻿An increased risk for JIA was found in children who had breast fed for less than 4 months, as opposed to those who were continued on breast milk beyond 4 months of age (aOR 3.5, 95% CI 1.4-8,5; *p* = 0.006). ﻿A short duration of exclusive as well as total breastfeeding was associated with an increased risk of JIA (aOR 1.3, 95% CI 1.1-1.6; *p* = 0.008 and aOR 1.2, 95% CI 1.1-1.3; *p* < 0.001). All associations between breastfeeding and JIA persisted after adjustment. There was no relationship between early nutrition and non-chronic arthritis.

**Conclusions:**

Our results indicate that there are different disease mechanisms for different types of arthritis in childhood. Longer duration of breastfeeding (both total and exclusive) may protect against development of JIA. Mothers should be encouraged to breast-feed their babies exclusively, if at all possible, for 4 months and continue partial breastfeeding for an extended time when foreign proteins are introduced.

## Background

Juvenile idiopathic arthritis (JIA), the most common chronic rheumatic disease of childhood, is a collection of chronic pediatric arthritis characterized by onset before 16 years of age and the presence of arthritis (inflammation of the synovium with thickening of the synovial lining and accumulation of synovial fluid) for at least 6 weeks [1].

JIA is considered to be an autoimmune disease, which is a result of an immune reaction caused or triggered by environmental factors in a genetically susceptible individual. The starting point of autoimmunity leading to this disease is still unknown.

The mother’s immunological memory is transferred to her infant via breast milk, and breast milk contains a variety of immune-modulating compounds, both immune cells and their products such as cytokines. Breastfeeding leads to immunological imprinting and programming of the infant [2], and thereby contributes to the maturity of the infant’s immune system [3–5]. It has been proposed that breastfeeding might protect against the development of JIA [6]. Another study noted a tendency of shorter breastfeeding among children who later developed oligoarticular JIA [7]. However, breastfeeding did not show any protective effect against JIA in subsequent studies [8–10]. A cohort study from UK recently showed that breastfeeding is associated with milder onset of JIA [11]. Previous studies have been retrospective case-control studies. To get more reliable unbiased results, we have analyzed data on early feeding of children in a prospective cohort study of the general population, the ABIS study (All Babies in Southeast Sweden). The primary aim of this study was to explore feeding factors, such as breastfeeding, in relation to risk of later development of JIA. Increased knowledge of the role of early nutrition of the child and its association with autoimmunity is of great importance for public health, as dietary recommendations may help to prevent these chronic diseases.

## Methods

In Sweden, all children aged 0-18 years with diagnosed JIA or Rheumatoid Arthritis (RA) are treated at pediatric clinics in hospitals or pediatric rheumatology clinics. The Swedish National Patient Register is maintained by the Swedish National Board of Health and Welfare (http://www.socialstyrelsen.se/english). This population-based register, was launched in 1964, but complete coverage of all in-patient care in Sweden did not begin until 1987 [12]. Currently, more than 99% of all somatic and psychiatric hospital discharges are registered. Since 2001 the register also covers outpatient visits from both private and public caregivers. Diseases are coded using International Classification of Diseases (ICD) codes and for each healthcare contact several items are recorded, including personal identity number (PIN - a unique 10-digit number assigned to all Swedish residents) [13], date of admission and discharge, hospital and primary and secondary diagnoses.

The Swedish JIA-registry started in 2009 with the primary goal to follow all children on cytokine modulators, but later expanded to all children with JIA. In 2014 there were 1700 patients included and coverage was almost complete. Through the unique PIN, information on each individual patient can be linked to other registries.

### Participants and design

All parents with children born between October 1,1997 and October 1,1999 in Southeast Sweden (*n* = 21,700) were asked to participate in the ABIS study, a prospective cohort study. 17,055 (78.6%) out of the 21,700 families who were asked gave their informed consent to participate. Questionnaires were completed at birth and then at the ages 1, 2.5, 5, 8 and 11-13. Different biological samples were collected. A diary was used for daily registration of certain facts related to nutrition, infections etc. during the first year of life. In the questionnaires, parents were asked for the duration of breastfeeding. Exclusive breastfeeding is defined as the infant receiving only breast-milk. Partial breastfeeding is defined as breastfeeding in addition to formula or other food (corresponding to WHO’s definition of complementary feeding) [14]. Total duration of breastfeeding is defined as the length of any kind of breastfeeding.

We also have considered a possible influence by the introduction of infant formula or gruel, cow’s milk and gluten, heredity, mode of delivery, and various socioeconomic variables such as civil status, parental age, education, smoking habits, and whether or not the parents were born outside Sweden. Questions pertaining to the duration of exclusive breastfeeding and total duration of breastfeeding, as well as the time for introduction of formulas and other semi-solid (infant formula, follow-on formula) and solid foods (porridge, first solid food) had response alternatives of months (after birth), usually from 1 to ≥9. The 1 year questionnaires were completed by 10,883 families, and in addition a detailed diary during the first year of life, including the exact time (date) of weaning and introduction of different food items, was collected for 9849 children. All subjects with missing values were excluded from the statistical model.

Using the Swedish personal identification number [13], we linked data between the ABIS-cohort and The Swedish National Patient Register [12]. Diagnoses are classified according to the ICD, versions 8 to 10. We identified 59 children, born between October 1, 1997 and October 1, 1999 in the ABIS region, with inpatient hospital discharges or outpatient visits who had a diagnostic code of JIA (M08-09 on ICD 8-10) and had accepted to participate in ABIS. After communication with all pediatric rheumatologists at local hospitals who reviewed the medical records of all 59 patients, 17 patients were excluded due to misdiagnosis (mostly arthritis that later proved to be reactive arthritis). Three of the 59 children had moved out of the ABIS region, but could be found with the PIN. Of the remaining 42 children, 41 guardians had completed the screening questionnaire after delivery, and 32 of them had completed both the screening questionnaire after delivery and the 1 year follow up questionnaire. 29 of 32 had also completed the diary during the first year. All cases of JIA were controlled via The Swedish pediatric JIA-registry.

We identified 111 patients in the same cohort with an episode of a transient non-chronic arthritis, with only one joint involved with a maximum duration of 6 weeks. 12% (13/111) of them were diagnosed with reactive arthritis and 88% (95/111) with unspecified arthritis (M02 respective M13 on ICD 8-10). The risk of rheumatic disease is considered to be minimal in this group, with only one period of monoarthritis with a short duration throughout the 17-19 year-long follow up period.

Data were analyzed as a case-control study with two groups of cases (JIA and non-chronic arthritis) and remaining children as controls.

### Statistics

The normality for independent variables was revised both graphically and by the Shapiro–Wilk test and, subsequently, homogeneity of variance was tested using Levene’s test.

Univariate logistic regression was used to calculate odds ratio (OR) and 95% confidence intervals (95% CIs). We used JIA as the dependent variable. Duration of breastfeeding (continuous as months from the 1-year questionnaire and as days from the diary), age of introduction of food (continuous as months) and possible confounding factors (listed below) associated with incidence of JIA were analyzed. In the second statistical model we used the same covariates but changed the dependent variable to non-chronic arthritis. Duration of breastfeeding was also dichotomized to shorter or longer than 4 and 6 months respectively, according to the recommendation to introduce solid food during 4 to 6 months. Adjusted odds ratio (aOR) was calculated in the final multivariable logistic regression model. Variables with *p* < 0.1 in the univariate analyses were included in the adjusted model as possible confounding factors associated breastfeeding. Attributable risk percent was calculated using the formula two in Rockhill et al. [15].

All *P*-values are two-tailed. A *P*-value below 0.05 and a 95% CI not overlapping the null value 1.00 for the OR were regarded as statistically significant.

Possible confounding factors were: heredity for rheumatism (JIA or RA in first- and second-degree relatives), parity, mode of delivery, preterm birth, introduction of formula,

parent-reported infections during child’s first year of life (cold, fever, throat infection/tonsillitis, influenza, otitis media, pneumonia, measles, rubella, mumps, pertussis, varicella, urinary tract infection, gastroenteritis and other infection), mother’s civil status, parental age, education, smoking habits, and whether or not the parents were born outside Sweden.

Statistics were calculated using the Statistical Package for Social Science (SPSS 20.0 software; SPSS Inc., Chicago, IL, USA).

## Results

### Cases

32 cases of JIA with complete questionnaires after delivery and at the 1 year follow up were identified. The most common JIA category was oligoarticular disease (Table 1). The unclassified based on the ILAR-criteria [1], either not fulfill criteria for any category or they meet categorization for 2 subtypes. Family history of RA/JIA in first-degree family member was present in only one patient in both the JIA-group (3%) and the group with non-chronic arthritis (1%) compared with 130 controls (1%) (Table 1). A family history of RA/JIA in a second-degree family member was more common; three patients in the JIA-group (9%), 16 in the group with non-chronic arthritis (14%), and 940 of the controls (9%).

**Table 1:** Risk of JIA and non-chronic arthritis according to early feeding and family history. *P*-values from logistic regression. Classification according to ILAR criteria. The number (n) and proportion (%) of patients in each category

		Controls	Non-chronic arthritis			JIA				
			Univariate analysis^a^ vs controls			Univariate analysis vs controls			Adjusted for potential confounding factors^b^	
		(*n* = 10,883)	(*n* = 111)	OR (95% CI)	p	(*n* = 32)	OR (95% CI)	p	aOR (95% CI)	p
Duration of exclusive breastfeeding										
	Mean (SD) months	4.5 (1.9)	4.6 (1.9)			3.6 (1,6)				
	Median (Range) months	4 (1 - 9)	4 (1 - 9)	1.0 (0.9-1.1)	0.879	4 (1 - 7)	1.3 (1.1-1.6)	**0.004**	**1.3 (1.1-1.6)**	**0.008**
	Mean (SD) days^c^	126 (60)	135 (61)			109 (55)	1.0 (1.0-1.0)	**0.005**	**1.0 (1.0-1.0)**	**0.009**
	Exclusive breastfeeding <4 months (n)	5351 (52.3%)	61 (51%)	1.3 (0.9-2.1)	0.220	22 (73%)	2.5 (1.2-5.6)	**0.026**	**2.5 (1.2-5.6)**	**0.029**
Duration of total breastfeeding										
	Mean (SD) months	7.1 (2.4)	7.2 (2.4)			5.7 (2.8)				
	Median (Range) months	8 (1 - 9)	8 (1 - 9)	0.99 (0.9-1.1)	0.940	6 (1 - 9)	1.2 (1.1-1.4)	**<0.001**	**1.2 (1.1-1.3)**	**<0.001**
	Mean (SD) days^d^	219 (82)	225 (90)	0.97 (0.9-1.1)	0.982	166 (100)	1.0 (1.0-1.0)	**<0.001**	**1.0 (1.0-1.0)**	**<0.001**
	Total breastfeeding <4 months (n)	1694(16%)	19 (18%)	1.1 (0.7-1.8)	0.715	11 (36%)	2.8 (1.3-5.8)	**0.006**	**2.8 (1.3-5.7)**	**0.009**
	Total breastfeeding <6 months (n)	3166 (31%)	33 (31%)	1.0 (0.7-1.5)	0.981	19 (61%)	3.6 (1.7-7.4)	**<0.001**	**3.5 (1.6-6.2)**	**<0.001**
Introduction of cow’s milk formula										
	Mean (SD) months	4.8 (2.5)	4.9 (2.7)	1.0 (0.9-1.1)	0.770	3.9 (2.2)	1.2 (1.0-1.3)	0.052	1.0 (1.0-1.3)	0.055
	Median (Range) months	5 (1 - 9)	6 (1 - 9)			4 (1 - 9)				
Introduction of gluten										
	Mean (SD) months	6.0 (1.39)	5.9 (1.4)	1.0 (0.9-1.2)	0.563	5.8 (1.27)	1.1 (0.9-1.4)	0.483	No analysis performed	
	Median (Range) months	6 (1 - 9)	6 (3 - 9)			6 (4 - 8)				
Family history / Heredity for JIA/RA										
	Mother with RA (n)	100 (1%)	1 (1%)	0.9 (0.3-6.7)	0.941	1 (3%)	3.5 (0.5-2.6)	0.223	No analysis performed	
	Father with RA (n)	31 (0%)	0 (0%)	0	1.000	0 (0%)	0	1.000	No analysis performed	
	Siblings with JIA/RA (n)	0 (0%)	0 (0%)	0	1.000	0 (0%)	0	1.000	No analysis performed	
	2nd degree family member with RA (n)	940 (9%)	16 (14%)	1.7 (0.98-2.8)	0.06	3 (9%)	1.7 (0.98-2.8)	0.910	No analysis performed	
Category at Onset (ILAR) (n)										
	Oligoarticular^a^					14 (44%)				
	Polyarticular^e^					4 (13%)				
	Enthesitis-related arthritis					2 (6%)				
	Psoriatic arthritis					3 (9%)				
	Systemic					4 (13%)				
	Unclassifiable					5 (16%)				

^a^ Patients with oligoarticular disease, not possible to classify as extended or persistent because of missing data

^b^ Potential confounding factors were heredity for rheumatism (JIA or RA in first- and second-degree relatives), parity, mode of delivery, preterm birth, infections during the first year of life, introduction of formula, the parents’ level of education, smoking and age as well as country of birth

^c^Data from diary: Control (*n* = 8913); Non-chronic arthritis (*n* = 79); JIA (*n* = 29)

^d^ Data from diary: Control (*n* = 9732); Non-chronic arthritis (*n* = 105); JIA (*n* = 31)

^e^ Patients with polyarticular disease, not possible to classify as RF positive or negative because of missing data

### Exclusive breastfeeding

**Fig. 1:**
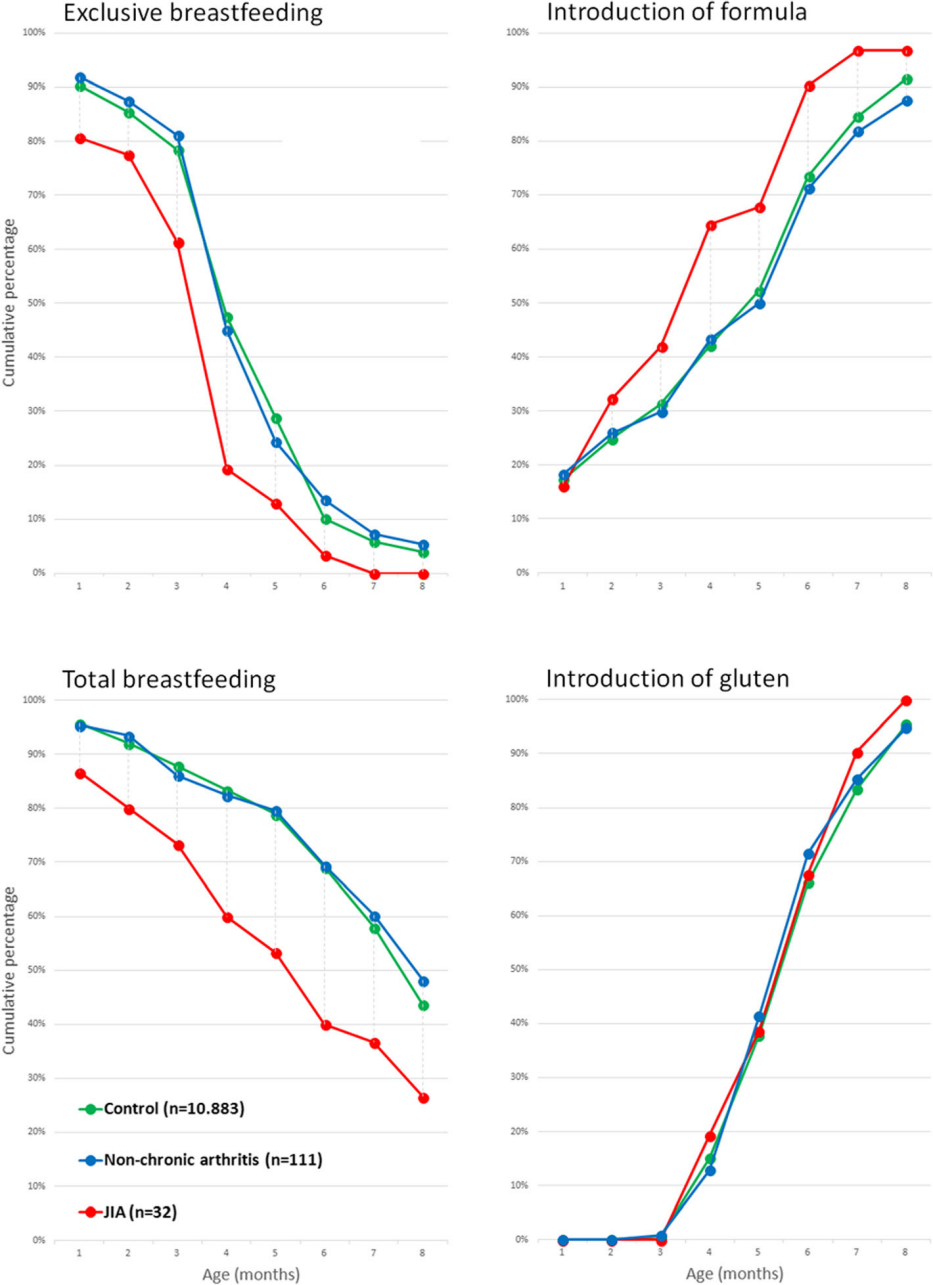
Cumulative percentages for duration of exclusive and total breastfeeding, as well as cumulative percentages for time of introduction of formula and gluten

One-year post-birth questionnaire data from the whole ABIS cohort 1 year after birth showed that the mean duration of exclusive breastfeeding was four and a half months, the same for boys and girls. The corresponding mean in the JIA-group was three and a half months. At the age of 3 months 78.4% of the unaffected children were exclusively breastfed compared to 61.3% in the affected (JIA) group (Fig. 1). A short duration of exclusive breastfeeding was significantly associated with an increased risk of JIA in logistic regression with continuous variables, both by months (from the one-year post-birth questionnaire) and by days (from the diary).

Dichotomized, exclusive breastfeeding less than 4 months, had highest statistical significance, associated with increased risk of developing JIA (aOR 2.50, 95% CI 1.20-5.56, *p* = 0.029), and attributable risk percent at 60.02%. Stratified data for end of breastfeeding are given in Table 2.

All associations between exclusive breastfeeding and JIA remained statistically significant when factors such as parental smoking, parents´ education, parental country of birth, parental age, heredity for rheumatism, preterm birth and introduction of formula and infections during the first year of life were included in the analysis (aOR 1.30, 95% CI 1.07-1.57; *p* = 0.008).

The group with non-chronic arthritis had the same mean (4.55 months) as the general population and there was no significant association between exclusive breastfeeding and non-chronic arthritis.

### Risk factors associated with short-term exclusive breastfeeding

The main logistic regression showed that short-term exclusive breastfeeding was positively associated with older maternal age (aOR 1.42, 95% CI 1.25-1.42), paternal age (over 37 years) (aOR 0.74; 95% CI 0.55–0.99; *p* = 0.045), maternal BMI >30 (aOR 1.07; 95% CI 1.05–1.09; *p* < 0.001) and with maternal smoking (aOR 1.43; 95% CI 1.05–1.95; *p* = 0.023). Short-term exclusive breastfeeding was more often reported by single parents (aOR 2.10; 95% CI 1.43–3.09; *p* < 0.001).

Short-term exclusive breastfeeding was less common if one of the parents had a university degree (Mother: aOR 0.74; 95% CI 0.61–0.90; *p* = 0.003, Father: aOR 0.73; 95% CI 0.58–0.92; *p* = 0.008).

### Total duration of breastfeeding

The mean duration of total breastfeeding was 1.5 months less in the affected children than the unaffected children (5.65 v. 7.09 months). The percentages of control infants who were breastfed at 3, 6 and ≥9 months of age were 87.9, 69.1 and 43.6, respectively. The corresponding percentages for any breastfeeding in the JIA-affected group were 73.3, 40.0 and 26.7 (Fig. 1). A short duration of total breastfeeding was associated with an increased risk of JIA in logistic regression with continuous variables.

Total duration of breastfeeding less than 4 months was associated with increased risk of developing JIA (aOR 2.75, 95% CI 1.33-5.71, *p* = 0.009), with a attributable risk percent at 64,17%.

The adjusted odds ratio for increased risk of JIA was 3.5 (95% CI 1.6-6,2; *p* = 0.001) if the total duration of breastfeeding stopped at or before 6 months of age, with a attributable risk percent at 71.97%.

Estimates between total duration of breastfeeding and JIA remained significant after adjusting for potential confounders including parental smoking, education, parental country of birth, parental age, heredity, preterm birth, introduction of formula, and infections during the first year of life (aOR 1.20, 95% CI 1.07-1.34 *p* < 0.001).

Non-chronic arthritis had the same mean duration (7.15 months) as the general population and there were no significant associations.

### Risk factors associated with total duration of breastfeeding

Both maternal (*P* < 0.001) and paternal age (*P* < 0.001) were positively associated with the total duration of breastfeeding. Short-term exclusive breastfeeding was more often reported by younger mothers.

### Introduction of cow’s milk formula

According to the questionnaire at 1 year, the mean age of introduction of formulas containing cow’s milk was 1 month less in the JIA affected group than the unaffected (3.94 v. 4.82 months) (Fig. 1). In logistic regression analysis, early introduction of cow’s milk formula (before 4 months of age) was associated with an increased risk of developing JIA (OR 2.49, 95% CI 1.19-5.21; *p* = 0.015). There was only a tendency of association between introduction of cow’s milk and JIA (aOR 0.87, 95% CI 0.997-1.32; *p* = 0.055) in logistic regression with continuous variables.

**Table 2:** End of breastfeeding comparison between JIA and Non-chronic arthritis vs. Controls. OR and *P*-values from logistic regression

		Controls	Non-chronic arthritis			JIA		
		(*n* = 10,189)	(*n* = 105)	OR (95% CI)	p	(*n* = 29)	OR (95% CI)	p
End of exclusive breastfeeding								
	Months 1-4 (0-120 days)	3261 (37%)	27 (34%)	1 (ref)	0.700	16 (57%)	1 (ref)	**0.023**
	Months 5-8 (121-240 days)	5374 (60%)	48 (61%)	0.9 (0.6-1.3)	0.458	12 (43%)	0.3 (0.1-0.7)	**0.006**
	Months 9-12 (241-365 days)	269 (3%)	4 (5%)	1.1 (0.5-2.8)	0.800	0 (0%)	0	0.994
End of total breastfeeding								
	Months 1-4 (0-120 days)	1579 (15%)	20 (19%)	1 (ref)	0.558	11 (38%)	1 (ref)	**0.003**
	Months 5-8 (121-240 days)	3569 (35%)	34 (32%)	0.8 (0.4-1.3)	0.315	11 (38%)	0.4 (0.2-1.0)	0.056
	Months 9-12 (241-365 days)	5041 (50%)	51 (49%)	0.8 (0.5-1.3)	0.397	7 (24%)	0.2 (0.1-0.5)	**0.001**

Non-chronic arthritis had the same mean (4.92 months) as the general population and there were no significant associations.

The age at introduction of formulas containing cow’s milk correlated negatively with both duration of exclusive breastfeeding (r 0.6, *p* < 0.001) and total duration of breastfeeding (r 0.575, *p* < 0.001).

### Introduction of gluten

We did not observe any significant association between age at introduction of gluten and risk of JIA or non-chronic arthritis (Fig. 1).

## Discussion

In the present study, we found that a short total duration of breastfeeding as well as a short duration of exclusive breastfeeding was associated with an increased risk of JIA. An early introduction of formula (<4 months of age) was associated with an increased risk of JIA. All associations remained statistically significant when potential confounders were included in the model.

Children with non-chronic arthritis had the same pattern of breastfeeding and introduction of formula as the general population. This suggests different disease mechanisms for different types of arthritis in children. Breastfeeding appears to be involved in the origin of autoimmunity causing Juvenile idiopathic arthritis.

### Breastfeeding data

The average breastfeeding duration in our study was similar to that among infants in Norway [16], Iceland [17] and Denmark [18], showing a relatively high prevalence of breastfeeding in Scandinavia compared with other parts of the Western world [19–21].

The data on total duration of breastfeeding is in good agreement with data from Official Statistics of Sweden [22] for the same area during the study period 1997–1999. Data is also consistent with the breastfeeding prevalence in the entire country during the years 1997–1999 (The National Board of Health and Welfare, 2001, . This indicates that the frequency of breastfeeding in ABIS infants during 1997–1999 was in line with current national recommendations, i.e. almost 85% of infants were breastfed until 4 months of age and more than 90% were introduced to solid food during the recommended 4 to 6 months.

### Study strengths and limitations

Earlier studies on the association between breastfeeding and JIA/JCA are all retrospective, and the results may be compromised by recall bias. Mason et al. [6] showed that children with Juvenile Rheumatoid Arthritis were less likely, then their matched controls, to have been breastfed. This could not be confirmed in subsequent studies [8–10]. However, these reports have mainly excluded children with Enthesitis-related arthritis and Psoriatic arthritis. In a relatively large study, Hyrich et al. recently showed that breastfeeding is associated with milder onset of JIA [11]. This, in addition to our results, provides the most reliable evidence so far for an association between breastfeeding and JIA. Breastfeeding may be associated with lesser occurrence, milder or delayed onset of this autoimmune disease.

Our study has several notable strengths. First, our prospective study design avoids the potential recall and selection biases of retrospective, case-control studies which collect data on diet, nutrition, feeding practice and lifestyle after the diagnosis of JIA. Second, all cases of JIA collected through the unique 10-digit PIN with codes from the Swedish National Patient Register have been confirmed via The Swedish pediatric JIA-registry and the medical records, a significant advantage over studies that rely on self-report. Third, the availability of detailed information on parental smoking, socio economic factors, parental age and heredity, and other important early life factors allowed us to control for a number of potential confounding factors that may have influenced our observed associations. Finally, we have detailed information regarding other potential early life factors, including infectious diseases and early exposure to antibiotics, which may also influence risk of JIA.

Even though we find significant correlations one has to be cautious in the conclusions. A limitation of our study is the small patient sample, due to the nature of the study i.e. a birth cohort of a relative rare disease. There are large variations in the clinical severity of the disease between the JIA categories. Therefore, different categories of JIA should be studied as separate groups [23, 24]. The patient sample in this study was too small to allow subgroup analysis.

Another weakness of the current study could be that the nutritional data was collected from the questionnaire at 1 year after birth, and there is a risk of recall bias [25]. However, this risk should not be overestimated as Launer et al. [26] have shown that mothers may well be aware of the breastfeeding duration of their latest child. When we compare breastfeeding data from the 1 year questionnaires and from the diary, the durations were comparable, indicating minimal risk of recall bias.

Although this is a cohort study we have chosen to analyze it with a case-control design since the exposure, duration of breastfeeding, is a continuous variable.

### Possible mechanisms underlying the protective effect of breastfeeding

Socioeconomic status affects the duration of breastfeeding, as was shown in our study. However, the relationship between breastfeeding and JIA persisted after adjusting for socioeconomic differences.

In Sweden, most children receive cow’s milk formula as their first source of foreign protein. The early introduction of cow’s milk formula correlated significantly with both the duration of exclusive breastfeeding and the duration of total breastfeeding, which is a confounding factor in this kind of study [27]. However, the statistical analysis with logistic regression shows that exclusive duration of breastfeeding (OR 1.28) and adjusting for introduction of cow’s milk formula (OR 1.32), indicating that breastfeeding has an independent protective effect against JIA.

Introduction of foreign proteins during breastfeeding favors the development of oral tolerance [28, 29]. Breast milk supports the development of tolerance [30–32]. Furthermore, breast milk is a source of other bioactive components such as hormones, immunoglobulins, enzymes and anti-inflammatory agents, which probably stimulates the development of the immune system [31, 32].

Breastfeeding could be linked indirectly to JIA through the gut microbiota of the infant. Gut bacteria play a fundamental role in the human body by promoting intestinal homeostasis [33–36]. The dysbiosis of the gut microbiota has been associated to an increasing number of diseases, including autoimmune diseases such as diabetes and inflammatory bowel disease [35, 37, 38].

Breastfeeding has been reported to influence infant gut microbiota development [39–41]. Breast milk is recognized as one of the most important postpartum elements modulating metabolic and immunologic programming in the child [42]. Formula-fed infants have increased richness of species compared with breastfed infants [43]. However, a fully developed bacterial flora is established within the intestinal tract only with the start of solid food consumption. [44–47].

## Conclusions

We cannot point to any specific mechanism effect, and we call for caution as the population-based finding is difficult to transfer to the individual situation. Still, the conclusion of our results is that a short duration of both total and exclusive breastfeeding seems to be associated with an increased risk of JIA in an unselected population of Swedish children. This indicates that breastfeeding might protect against development of JIA. Mothers should be encouraged to exclusively breastfeed their babies, if at all possible, for at least 4 months and continue partial breastfeeding during the time when foreign proteins are introduced via food.

## Abbrevations

ABIS: All Babies in Southeast Sweden
aOR: Adjusted odds ratio
CI: Confidence Interval
ICD: International Classification of Diseases
ILAR: International League of Associations for Rheumatology
JCA: Juvenile Chronic Arthritis
JIA: Juvenile Idiopathic Arthritis
JRA: Juvenile Rheumatoid Arthritis
OR: Odds Ratio
PIN: Personal Identification Number
RA: Rheumatoid Arthritis
RR: Relative risk
